# Psychometric properties of the Brief Symptom Inventory support the hypothesis of a general psychopathological factor

**DOI:** 10.47626/2237-6089-2021-0207

**Published:** 2021-03-31

**Authors:** Alexandre Luiz de Oliveira Serpa, Danielle Souza Costa, Clarice de Medeiros Chaves Ferreira, Mayra Isabel C. Pinheiro, Alexandre Paim Diaz, Jonas Jardim de Paula, Debora Marques Miranda, Antônio Geraldo da Silva, Leandro Fernandes Malloy-Diniz

**Affiliations:** 1 Laboratório de Neurociências Cognitiva e Social Universidade Presbiteriana Mackenzie São Paulo SP Brazil Laboratório de Neurociências Cognitiva e Social, Universidade Presbiteriana Mackenzie, São Paulo, SP, Brazil.; 2 Metacognitiv São Paulo SP Brazil Metacognitiv, São Paulo, SP, Brazil.; 3 Laboratório de Neurociências Faculdade de Medicina Universidade Federal de Minas Gerais Belo Horizonte MG Brazil Laboratório de Neurociências, Faculdade de Medicina, Universidade Federal de Minas Gerais (UFMG), Belo Horizonte, MG, Brazil.; 4 Universidade FUMEC Belo Horizonte MG Brazil Universidade FUMEC, Belo Horizonte, MG, Brazil.; 5 SGTES Ministério da Saúde Brasília DF Brazil Secretaria de Gestão do Trabalho e da Educação na Saúde (SGTES), Ministério da Saúde, Brasília, DF, Brazil.; 6 Department of Psychiatry and Behavioral Sciences The University of Texas Health Science Center at Houston Houston TX USA Louis A. Faillace, MD, Department of Psychiatry and Behavioral Sciences, The University of Texas Health Science Center at Houston, Houston, TX, USA.; 7 Departamento de Psicologia Faculdade de Ciências Médicas de Minas Gerais Belo Horizonte MG Brazil Departamento de Psicologia, Faculdade de Ciências Médicas de Minas Gerais, Belo Horizonte, MG, Brazil.; 8 Departamento de Pediatria Faculdade de Medicina UFMG Belo Horizonte MG Brazil Departamento de Pediatria, Faculdade de Medicina, UFMG, Belo Horizonte, MG, Brazil.; 9 Associação Brasileira de Psiquiatria Rio de Janeiro RJ Brazil Associação Brasileira de Psiquiatria, Rio de Janeiro, RJ, Brazil.; 10 Universidade do Porto Porto Portugal Universidade do Porto, Porto, Portugal.; 11 Departamento de Saúde Mental Faculdade de Medicina UFMG Belo Horizonte MG Brazil Departamento de Saúde Mental, Faculdade de Medicina, UFMG, Belo Horizonte, MG, Brazil.

**Keywords:** Psychological factors, reproducibility of results, mental disorders, psychometrics

## Abstract

**Introduction:**

The existence of a general factor related to psychiatric symptoms is supported by studies using a variety of methods in both clinical and non-clinical samples.

**Objectives:**

This study aims to evaluate the replicability of the internal structure of the Brief Symptom Inventory in a large Brazilian sample.

**Methods:**

Participants were 6,427 Brazilian subjects (81% female). Mean age was 42.1 years (standard deviation [SD] = 13.6, Min = 13, Max = 80). All participants completed the online version of the Brief Symptom Inventory. This scale presents a general score (GSI) and nine specific clusters of symptoms (depression, anxiety, phobic anxiety, interpersonal sensibility, psychoticism, paranoid ideation, obsessive-compulsive behavior, hostility, and somatization symptoms).

**Results:**

Confirmatory factor analysis was performed to assess the factor structure of the BSI. The results showed that the best-fitting model was a bifactor solution and the general factor was the main dimension explaining most of the reliable variability in the data.

**Conclusion:**

The findings suggest that the BSI’s internal structure was replicated in a non-clinical sample and that the general factor is the most reliable score. However, it is necessary to better understand the meaning of the general factor scores in a non-clinical sample to increase interpretability of scores.

## Introduction

The frontiers between psychiatric illnesses are much less established than those conceived in the diagnostic manuals of mental disorders. The lack of precise boundaries between mental illnesses has modified the process of psychiatric diagnosis, with a gradual change from a categorical perspective to a dimensional one.^1^ Current categories of mental disorders are highly comorbid with each other and this phenotypic covariance should not be neglected in clinical practice. Some authors even argue that the symptomatic similarity between patients with specific disorders suggests a shared common core between mental disorders.^2,3^

Recently, Caspi and Moffitt^4^ pointed out that several psychopathologies often have the same biomarkers and risk factors and that therapeutic strategies seem to work for a broad range of disorders. Therefore, regardless of their nosological classification, assessment of psychopathological symptoms has significant clinical applicability to identify therapeutic targets and to assess the clinical relevance of interventions. Carragher et al.^5^ even reason that symptom-level analyses allow us to “unpack disorders,” uncovering the empirically (nonarbitrary) based structure of psychopathology. Their study found a modified bifactor model with three correlated specific factors (internalizing, externalizing, thought disorder) and one general psychopathology factor, the “p” factor. The “p” factor has since been corroborated by studies with children and adolescents^6^ and with adults,^2^ a pattern that is likely to be stable over time.^6,7^ In effect, if the “p” factor is not an erroneous finding, it will consistently appear in the psychometric modeling of instruments measuring different types of psychopathological dimensions. Therefore, as in studies concerning constructs like the general intelligence factor, also called “g,” psychometric analyses of instruments evaluating psychiatric disorders or psychological distress are a useful way to assess the hypothesis of the existence of a “p” factor.

The Brief Symptom Inventory (BSI)^8^ is a self-report instrument developed to assess psychological distress and psychopathological symptoms in nine dimensions: depression, anxiety, somatization, obsession-compulsion, interpersonal sensitivity, phobic anxiety, hostility, paranoid ideation, and psychoticism. The inventory also produces a Global Severity Index (GSI), which includes all symptoms assessed by the scale. The scale was developed before the third edition of the Diagnostic and Statistical Manual of Mental Disorders (DSM-III)^9^ and has remained in use for clinical and research purposes with psychiatric patients and in non-clinical samples.^10,11^ The BSI has been translated into several languages in the decades since its development.^12-18^

The nine-factor structure of the BSI has been replicated through confirmatory and exploratory factor analysis in samples from countries like Italy^19^ and Azerbaijan,^20^ but a unidimensional factor structure has also been found in other countries, like the United States^21^ and Greece.^22^ These inconsistencies are usually due to the communality of items measuring psychiatric symptoms, which could vary between samples from different cultures. Thomas^23^ pointed out that these properties fit well within the classical conceptualization of bifactor models, as a result of their capacity to represent both unidimensional and specific factor variances at the same time, allowing identification of the single contribution of each source to the measured outcome, followed by an evaluation of BSI, considering its model. The bifactor model found fits the data better than the unidimensional and the correlated nine-factor models and also showed additional evidence of criterion-related validity by more accurately predicting DSM-IV-TR depression, generalized anxiety, phobic, and somatization disorders than those models. This result calls attention to the clinical relevance of the bifactor structure for explaining the variability in respondents’ responses. The bifactor structure was replicated by Urbán et al.^15^ in a Hungarian sample, and Urbán et al.^24^ in Hungarian and Dutch samples, demonstrating a better fit than other multidimensional and hierarchical structures. The bifactor structure was replicated for both men and women, the global scores were understood as a global distress factor, and the general factor seemed adequate to represent a global psychological distress index.

Nonetheless, the bifactor solution was not always found in other populations, as was the case in a non-clinical Greek sample.^22^ In this case, the authors argue that the BSI’s properties do not justify use of specific factors to assess psychopathology. However, even in that situation, the authors suggest that the BIS global score can act as a proxy of psychological distress in non-clinical samples.

The present study examined the latent factor structure of the BSI in a non-clinical Brazilian sample to: (a) replicate several plausible models reported in the literature; (b) compare these models to understand what best fits the Brazilian population; (c) provide evidence that allows the interpretability of BSI scores in regard to psychopathological symptoms; and (d) provide empirical evidence to contribute to the discussion of whether the unidimensional factor of BSI should be considered a reliable and valid representation of the “p” factor. These analyses are essential to understanding the BSI’s scores, strengths, and limitations and contribute to interpretation of the general and specific factors that could emerge from data.

## Method

### Participants

We included 6,427 (81% female) Brazilian subjects from all states of the country. The mean age was 42.1 years old (standard deviation [SD] = 13.6, Min = 13, Max = 90). In regard to educational level, 68% were classified from 6 to 8 on the International Standard Classification of Education – 2011,^25^ while 51% were married or in a stable relationship, and 2,468 (85.7% female) participants self-reported previous lifetime psychiatric diagnoses for at least one condition. All the participants included in the analyses gave their consent and had at least one valid answer for a BSI item.

### Procedure

Participants were recruited via the internet with a social media campaign run from May to June 2020, using a snowball sampling procedure. The SurveyMonkey platform delivered all the questionnaires. Participants gave informed consent before starting to answer the tests and questionnaires. Ethical procedures were approved by the National Research Ethics Commission (process CAAE 30823620.6.0000.5149) and comply with the Helsinki Declaration.

### Measure

The BSI is a 53-item instrument designed to identify relevant psychological symptoms.^8^ The inventory covers nine symptom dimensions (Somatization, Obsession-Compulsion, Interpersonal Sensitivity, Depression, Anxiety, Hostility, Phobic Anxiety, Paranoid Ideation, and Psychoticism) plus three global indices of distress (Global Severity Index – GSI, Positive Symptom Distress Index – PSDI, and Positive Symptom Total – PST). Items are answered on a 5-point Likert scale from 0 (not at all) to 4 (extremely). The instrument can be self-administered or interviewer-administered and has norms for adolescents up to 13 years old and for adults for both clinical and nonclinical groups. The reliability reported in the original manual ranged from 0.71 for Psychoticism to 0.85 for the Depression dimension. Test-retest reliability was demonstrated with global indices ranging from 0.87 (PSDI) to 0.90 (GSI) and for all dimensions, ranging from 0.68 (Somatization) to 0.91 (Phobic Anxiety).

### Statistical procedures

Five models were analyzed. Model 1 is the unidimensional model. Model 2 is the correlated nine-factor model, based on the factor structure of the BSI. Model 3 is the hierarchical model, comprising one second order (GSI) and nine first order factors with oblique correlations. Model 4 is the classical bifactor model, with one general factor and nine specific factors with correlations fixed at zero. And Model 5 is the exploratory bifactor model, with one general factor and nine specific factors free to correlate with each other.

A confirmatory factor analysis was conducted with the lavaan package^26^ in R software^27^ using weighted least squares mean and variance adjusted estimation with Satorra-Bentler correction, to correct the standard errors and chi-square estimates.^28^ Global model fit was evaluated using the comparative fit index (CFI), Tucker-Lewis index (TLI), and the root mean square error of approximation (RMSEA). To interpret model fit, values equal to or greater than 0.95 for CFI and TLI, and equal to or less than 0.05 for RMSEA were considered acceptable.^29^

The quality of the models was verified using several indices. The H index was developed to evaluate construct replicability, measuring the degree to which the indicators appropriately represent the latent variables. A threshold of 0.70 is generally accepted as a criterion for this index.^30^ Omega (ω) and omega hierarchical (ωH) coefficients were calculated. The omega hierarchical coefficient is useful for bifactor models for assessing the percentage of common variance attributable to the general factor. Reise et al.^31^ argue that the higher the omega hierarchical value, the higher the relevance of the general factor to explain the variance of the data. In that case, the general factor could reflect an essentially unidimensional structure that explains the variance in respondents’ scores.

To evaluate the unidimensionality of the factors, explained common variances were calculated for general (ECV), specific (ECV_SG and ECV_GS), and item levels (I-ECV). The ECV index evaluates the proportion of common variance explained by the general factor. The ECV_SG and ECV_GS indicate common variance explained related to specific factors and the variance in each factor due to the general factor, respectively. This indicates the proportion of the items’ variance that could be explained by the general factors.^30^ The percentage of uncontaminated correlations (PUC) specifies the possible data bias of interpreting multidimensional data into unidimensional data and PUC > 0.90 means that ECV, ω, and ωH can be interpreted directly. The semPlot^32^ and BifactorIndicesCalculator^33^ in R and Jamovi software^34^ were also used in these analyses.

## Results

Adequate solutions were found for all models and these results are presented in Table 1.

**Table 1:** Confirmatory factor analysis fit indexes and model comparison for the BSI

Model		S-Bχ^**2**^	df	CFI	TLI	RMSEA	RMSEA 90%CI
1	One factor	52166.585	1325	0.981	0.980	0.083	[0.082 - 0.083]
2	Nine correlated factors*	21190.698	1091	0.992	0.991	0.056	[0.055 - 0.056]
3	Hierarchical	30708.836	1316	0.989	0.988	0.063	[0.062 - 0.064]
*4*	*Bifactor*	*25602.890*	*1276*	*0.991*	*0.990*	*0.057*	*[0.057 - 0.058]*
5	Exploratory bifactor*	15822.885	1240	0.995	0.995	0.043	[0.042 - 0.044]
**Model comparison**		**Δχ** ^**2**^	**Δdf**				
Model 4							
Model 3		2848.7	40	p < 0.001			
Model 1		12643.8	9	p < 0.001			

* Latent variable covariance matrix not positive definite.

90%CI = 90% confidence interval; CFI = comparative fit index; df = degrees of freedom; RMSEA = root mean square error of approximation; S-B = Satorra-Bentler; TLI = Tucker-Lewis index.

Italic text indicates the best fitting model.

All models were statistically significant (p < 0.001).

However, for models 2 and model 5, where an oblique structure for the nine factors was allowed, the covariance matrix of latent variables was not positive definite, which suggests that the factor solution was unacceptable. For both models 2 and 5, this observation is probably because of the inflation of the correlation between the Depression and Psychoticism dimensions (Model 2: *r* = 1.047, p < 0.001; Model 5: *r* = 1.394). Examining the latent variable correlation matrix for Model 2, we found that around 66% of the correlations were equal to or greater than 0.750, indicating high interdependence of the latent variables. When controlling by the general factor in Model 5, around 42% of the correlations were not significant and the remaining correlations were above 0.750, except for the correlation between Depression and Psychoticism (Table 2).

**Table 2:** Correlation matrix between latent dimensions for Model 2, on the lower diagonal, and Model 5, on the upper diagonal

	S	OC	IS	D	A	H	PA	PI	P	GSI
S	1.00	0.22*	-0.12*	-0.17*	0.28*	-0.06^‡^	0.19*	0.03	-0.06	0.00
OC	0.75*	1.00	0.01	-0.05	-0.13*	0.06	0.01	0.08^‡^	0.06	0.00
IS	0.68*	0.83*	1.00	0.35*	-0.27*	0.25*	-0.06	0.74*	0.69*	0.00
D	0.70*	0.87*	0.91*	1.00	-0.15*	-0.01	-0.01	0.04	1.39*	0.00
A	0.78*	0.79*	0.77*	0.83*	1.00	-0.09†	0.57*	-0.25*	-0.08	0.00
H	0.63*	0.76*	0.82*	0.79*	0.77*	1.00	-0.05	0.33*	0.26*	0.00
PA	0.61*	0.61*	0.60*	0.65*	0.78*	0.54*	1.00	0.01	0.12^†^	0.00
PI	0.65*	0.75*	0.92*	0.78*	0.69*	0.78*	0.55*	1.00	0.58*	0.00
P	0.75*	0.89*	0.96*	1.05*	0.85*	0.84*	0.67*	0.91*	1.00	0.00
GSI										1.00

* p < 0.001, ^†^ p < 0.01, ^‡^ p < 0.05.

A = Anxiety; D = Depression; GSI = Global Severity Index; H = Hostility; IS = Interpersonal Sensitivity; OC = Obsession-Compulsion; P = Psychoticism; PA = Phobic Anxiety; PI = Paranoid Ideation; S = Somatization.

Models 1, 3, and 4 were therefore compared to determine the best-fitting model. All three models were adequate according to CFI and TLI, but Model 1 and Model 3 were inadequate because of high RMSEA values. A scaled chi-square difference also indicated that Model 4 is the best-fitting model. The standardized item parameters are presented in Table 3. Apart from item 3 (0.255), all items have factor loadings > 0.40 on the general factor. For all of the specific factors at least one item has a factor loading < 0.40, and for the Depression, Paranoid Ideation, and Psychoticism dimensions, just one item was above that level. The variance explained by the items from those three dimensions is strongly associated with the general factor, as demonstrated by I-ECV (Depression: Min = 0.884, Max = 0.985; Paranoid Ideation: Min = 0.609, Max = 0.819; Psychoticism: Min = 0.840, Max = 1.000).

**Table 3:** Standardized item parameter estimates, and explained common variance for the Bifactor model

Item	S	OC	IS	D	A	H	PA	PI	P	GSI	I-ECV
2	0.491									0.547	0.554
7	0.346									0.483	0.582
23	0.417									0.588	0.665
29	0.429									0.591	0.655
30	0.467									0.519	0.553
33	0.437									0.546	0.610
37	0.439									0.671	0.701
5		0.622								0.567	0.454
15		0.271								0.776	0.891
26		0.256								0.697	0.920
27		0.199								0.766	0.937
32		0.544								0.797	0.664
36		0.354								0.776	0.826
20			0.237							0.829	0.924
21			0.468							0.852	0.747
22			0.356							0.792	0.870
42			0.244							0.725	0.898
9				0.242						0.749	0.895
16				0.988						0.812	0.985
17				0.273						0.836	0.905
18				0.329						0.836	0.884
35				0.222						0.747	0.917
50				0.213						0.794	0.933
1					0.437					0.715	0.725
12					0.447					0.768	0.747
19					0.547					0.718	0.633
38					0.229					0.838	0.930
45					0.417					0.775	0.776
49					0.811					0.691	0.986
6						0.398				0.766	0.787
13						0.438				0.778	0.791
40						0.638				0.587	0.459
41						0.626				0.637	0.512
46						0.348				0.675	0.788
8							0.274			0.255	0.464
28							0.734			0.426	0.251
31							0.616			0.613	0.510
43							0.627			0.499	0.388
47							0.562			0.726	0.994
4								0.269		0.554	0.819
10								0.278		0.488	0.756
24								0.358		0.677	0.782
48								0.358		0.664	0.782
51								0.517		0.646	0.609
3									0.178	0.556	0.907
14									-0.375	0.859	0.840
34									0.244	0.673	0.884
44									-0.858	0.724	1.000
53									0.177	0.865	0.960
11										0.598	1.000
25										0.588	1.000
39										0.755	1.000
52										0.794	1.000

A = Anxiety; D = Depression; GSI = Global Severity Index; H = Hostility; I-ECV - Item explained common variance; IS = Interpersonal Sensitivity; OC = Obsession-Compulsion; P = Psychoticism; PA = Phobic Anxiety; PI = Paranoid Ideation; S = Somatization.

The general factor emerges as the central dimension to explain the variability of respondents’ answers. The H index indicates that the general factor achieved the best construct replicability whereas the other dimensions had low estimates, suggesting that they are not adequately defined, except for the Phobic Anxiety dimension (Table 4). For GSI, omega was 0.98 and omega hierarchical was 0.95, which suggests that around 97% of the reliable variance is due to the general factor, 3% is due to the specific factors, and 2% squarely to random error.^30^ For the specific factors, omega values range from 0.83 to 0.93 and are higher than their omega hierarchical values. These results suggest an essentially unidimensional structure as a result of a strong general factor that explains most of the reliable variance and is less affected by the multidimensionality induced by specific factors.

**Table 4:** Reliability, sources of variance, and replicability of the Bifactor Model for the BSI

	ECV_SG	ECV_GS	ω	ωH	Hr	PUC
S	0.04	0.62	0.87	0.33	0.62	
OC	0.03	0.77	0.92	0.19	0.56	
IS	0.01	0.85	0.91	0.13	0.34	
D	0.01	0.92	0.93	0.07	0.26	
A	0.03	0.79	0.94	0.18	0.54	
H	0.04	0.66	0.93	0.30	0.65	
PA	0.04	0.50	0.84	0.38	0.71	
PI	0.02	0.74	0.83	0.21	0.45	
P	0.01	0.91	0.87	0.00	0.23	
GSI	0.77	0.77	0.98	0.96	0.98	0.92

ω = Omega; ωH = Omega hierarchical; A = Anxiety; D = Depression; ECV_GS = Explained common variance– group to specific; ECV_SG = Explained common variance– specific to group; GSI = Global Severity Index; H = Hostility; Hr = construct replicability coefficient; IS = Interpersonal Sensitivity; OC = Obsession-Compulsion; P = Psychoticism; PA = Phobic Anxiety; PI = Paranoid Ideation; PUC = percentage of uncontaminated correlations; S = Somatization.

The ECV of the GSI explains 77% of the variance and, in conjunction with the PUC of 0.918, common variance might be interpreted as essentially unidimensional. Nevertheless, the comparison of ECV_SG and ECV_GS implies that most of the explained variance on the specific factors is due to the general factor and not to the item composition of the dimensions themselves. Also, most of the BSI items showed high communality by virtue of the GSI dimension, suggesting they are practical markers of measures of the general factor. Thus, evidence suggests that one general factor is sufficient to explain the score variability of the BSI.

**Discussion**

The present results provide replication of BSI internal structure models previously reported in different countries and samples. Five models were examined and the bifactor model was the best representation for Brazil. Our results strongly support the hypothesis of a unidimensional structure in the assessment of psychiatric symptoms using the BSI. They are in line with results previously reported,^15,23^ reinforcing the bifactor nature of the BSI regardless of cultural influences and mental health conditions. These results also support the hypothesis raised by Loutsiou-Ladd et al.^22^ suggesting that the BSI is unidimensional, at least in non-clinical samples.

Our results suggest that the general symptom index presents the most robust psychometric properties, rather than the specific factors. The idea of a “p” factor is supported by previous psychometric studies, which argue that a bifactor structure of symptoms explains most of the variability of presentation of psychopathological traits in the population. For example, Gluschkoff et al.,^7^ analyzed the results of interviews based on DSM criteria in a large non-clinical sample. They found that the clusters of symptoms related to specific diagnoses are explained by the bifactor structure with specific symptom clusters for mania, generalized anxiety disorder, major depressive disorder, dysthymia, posttraumatic stress disorder, agoraphobia, panic disorder, social phobia, specific phobia, antisocial personality disorder, distress, externalizing disorder, and internalizing disorder. Besides these specific factors, a general factor was also found. It is interesting to note that this bifactor structure remains relatively stable throughout the longitudinal follow up of the sample, suggesting that despite changes in symptom presentation, a general “p” factor continues to influence clinical presentation in psychopathology.

The “p” factor is also supported by its ecological relationship with health and educational indicators and behavioral problems. Recently, Pettersson et al.^35^ assessed a large population sample and also found that the general psychopathological factor, assessed by general scores derived from self and other-report psychiatric scales, was associated with some adverse outcomes of both prescribed and illegal drug use, criminality, and both low income and low educational level.

Our results present a bifactor structure for BSI, and the GSI emerges as the main factor for screening mental health in the general population, while the specific scores have little or no discriminatory power. Nonetheless, use of specific factors seems to be useful to address specific questions in both clinical and research settings, considering applications in diagnostic and treatment issues. For example, in recent studies concerning mental health related to pandemics, Wang et al.,^36^ and Ellis et al.,^37^ used hostility and depression subscales to address specific questions about psychological distress in cancer patients and adolescents in isolation, respectively. Thus, our results support a similar score interpretation approach to address specific mental health issues for the Brazilian version of the BSI.

Our study presents limitations that should be addressed by future studies. First of all, the gender imbalance in our sample may constitute a bias. Future studies are needed to assess gender invariance in BSI scores. The second limitation is that our sample was non-clinical and even though 38% of participants reported suffering from a psychiatric illness, we do not have in-depth assessments to verify this information. Nonetheless, our data are similar to those reported by Viana et al.^38^ in a Brazilian epidemiological study and, therefore, our sample can be considered similar to those previously studied in Brazil. The third limitation concerns the age range of our sample, which is very wide. Although it falls in line with our objective, which is to investigate the general factor structure of BSI for the Brazilian population, this might not capture or could mask differences in the course of intrapersonal development or those associated with individual or group niche characteristics.

As pointed out by Loutsiou-Ladd et al.,^22^ symptoms expressed in clinical samples can be different from non-clinical symptoms and, therefore, can affect the factor structure of symptom presentation. The fourth limitation is related to the period of data collection (from May to June 2020). Since the COVID-19 pandemic can impact the mental health of the population, increasing distress symptoms,^39^ the non-clinical characteristic of our sample is, to a certain extent, questionable. Therefore, future studies should assess whether this factor structure will remain relatively unchanged in a similar community sample in a post-pandemic scenario. Investigation of evidence of the validity of BSI scores is also important to understand exactly what they represent and how they might be interpreted.

Despite its limitation, our study reinforces the bifactor structure of psychiatric symptoms, as assessed by BSI, corroborating the accumulated evidence which suggests the existence of a general psychiatric factor independent from specific clusters of psychiatric symptoms. Future studies should discuss the interpretation of the unidimensional factor using the “p” factor approach in clinical samples and clinical settings, including the relationship with other symptom-based measures and the capacity to discriminate clinical samples in regard to their mental health disorders. The existence of a general indicator of global mental health would be a valuable tool for faster screening of individuals vulnerable to developing clinical conditions in large populations, providing them greater access to early healthcare and decreasing the costs of diagnosis and treatment and social problems associated with mental illness conditions.

**Figure 1:**
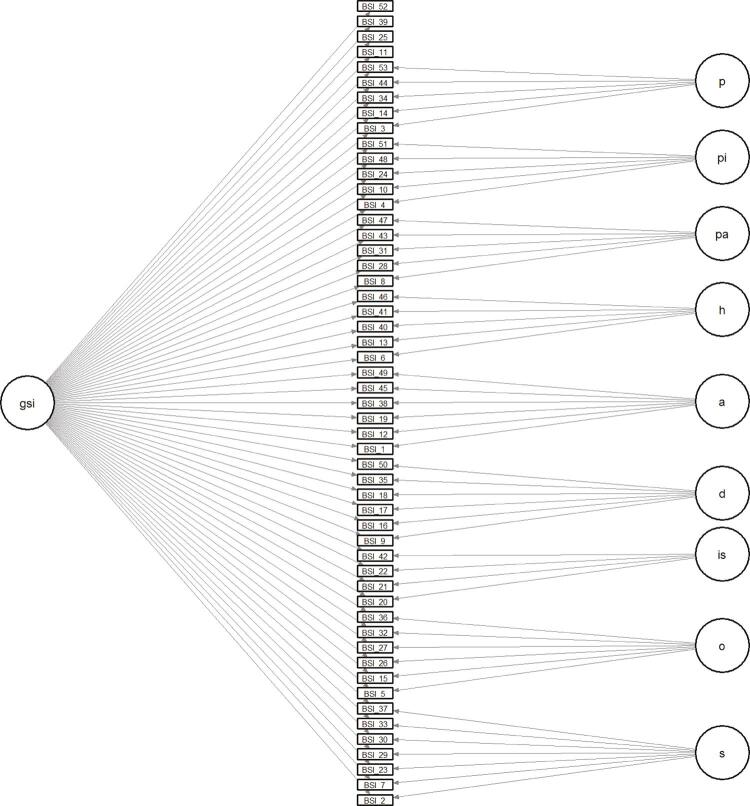
Graphical illustration of the bifactor model for BSI. A = Anxiety; D = Depression; GSI = Global Severity Index; H = Hostility; IS = Interpersonal Sensitivity; OC = Obsession-Compulsion; P = Psychoticism; PA = Phobic Anxiety; PI = Paranoid Ideation; S = Somatization.

## References

[1] . Haslam N, Holland E, Kuppens P. Categories versus dimensions in personality and psychopathology: a quantitative review of taxometric research. Psychol Med. 2012;42:903-20. 10.1017/S0033291711001966 21939592

[2] . Caspi A, Houts RM, Belsky DW, Goldman-Mellor SJ, Harrington H, Israel S, et al. The p Factor: one general psychopathology factor in the structure of psychiatric disorders? Clin Psychol Sci. 2014;2:119-37. 10.1177/2167702613497473 PMC4209412 25360393

[3] . Haltigan JD. Putting practicality into “p”: leveraging general factor models of psychopathology in clinical intervention. J Am Acad Child Adolesc Psychiatr. 2019;58:751-3. 10.1016/j.jaac.2019.03.005 30853501

[4] . Caspi A, Moffitt TE. All for one and one for all: mental disorders in one dimension. Am J Psychiatry. 2018;175:831-44. 10.1176/appi.ajp.2018.17121383 PMC6120790 29621902

[5] . Carragher N, Teesson M, Sunderland M, Newton NC, Krueger RF, Conrod PJ, et al. The structure of adolescent psychopathology: a symptom-level analysis. Psychol Med. 2016;46:981-94. 10.1017/S0033291715002470 26620582

[6] . Polek E, Neufeld SAS, Wilkinson P, Goodyer I, St Clair M, Prabhu G, et al. How do the prevalence and relative risk of non-suicidal self-injury and suicidal thoughts vary across the population distribution of common mental distress (the p factor)? Observational analyses replicated in two independent UK cohorts of young people. BMJ Open. 2020;10:e032494. 10.1136/bmjopen-2019-032494 PMC7223145 32398331

[7] . Gluschkoff K, Jokela M, Rosenström T. The general psychopathology factor: structural stability and generalizability to within-individual changes. Front Psychiatry. 2019;10:594. 10.3389/fpsyt.2019.00594 PMC6728891 31543833

[8] . Derogatis LR. BSI Brief Symptom Inventory: administration, scoring, and procedures manual. 4th ed. Minneapolis, MN: National Computer Systems. 1993.

[9] . American Psychiatric Association. Diagnostic and Statistical Manual of Mental Disorders, Third Edition (DSM-III). Washington: American Psychiatric Publishing; 1980.

[10] . Gavazzi SM, Julian TW, McKenry PC. Utilization of the Brief Symptom inventory to discriminate between violent and nonviolent male relationship partners. Psychol Rep. 1996;79:1047-56. 10.2466/pr0.1996.79.3.1047 8969115

[11] . Yasak Y, Esiyok B. Anger amongst Turkish drivers: Driving Anger Scale and its adapted, long and short version. Saf Sci. 2009;47:138-44.

[12] . Francis VM, Rajan P, Turner N. British community norms for the Brief Symptom Inventory. Br J Clin Psychol. 1990;29:115-6. 10.1111/j.2044-8260.1990.tb00857.x 2310864

[13] . Leo D, Frisoni GB, Rozzini R, Trabucchi M. Italian community norms for the Brief Symptom Inventory in the elderly. Br J Clin Psychol. 1993;32:209-13. 10.1111/j.2044-8260.1993.tb01045.x 8318938

[14] . Gilbar O, Ben-Zur H. Adult Israeli community norms for the Brief Symptom Inventory (BSI). Int J Stress Manag. 2002;9:1-10.

[15] . Urbán R, Kun B, Farkas J, Paksi B, Kökönyei G, Unoka Z, et al. Bifactor structural model of symptom checklists: SCL-90-R and Brief Symptom Inventory (BSI) in a non-clinical community sample. Psychiatry Res. 2014;216:146-54. 10.1016/j.psychres.2014.01.027 24524946

[16] . Jahn DR, DeVylder JE, Drapalski AL, Medoff D, Dixon LB. Personal recovery as a protective factor against suicide ideation in individuals with schizophrenia. J Nerv Ment Dis. 2016;204:827-31. 10.1097/NMD.0000000000000521 PMC5075268 27105456

[17] . Kwee CM, van den Hout MA. Anxiety sensitivity does not predict treatment outcome or treatment length in obsessive-compulsive disorder and related anxiety disorders. J Obsessive Compuls Relat Disord. 2019;21:18-25.

[18] . Boer S, Dekkers OM, Cessie SL, Carlier IV, van Hemert AM. Prediction of prolonged treatment course for depressive and anxiety disorders in an outpatient setting: the leiden routine outcome monitoring study. J Affect Disord. 2019;247:81-7. 10.1016/j.jad.2018.12.035 30658244

[19] . Adawi M, Zerbetto R, Re TS, Bisharat B, Mahamid M, Amital H, et al. Psychometric properties of the Brief Symptom Inventory in nomophobic subjects: insights from preliminary confirmatory factor, exploratory factor, and clustering analyses in a sample of healthy Italian volunteers. Psychol Res Behav Manag. 2019;12:145-54. 10.2147/PRBM.S173282 PMC6419603 30881158

[20] . Kerimova M, Osmanli N. The Brief Symptom Inventory: a validity-reliability Study of a Sample from Azerbaijan. J Educ Train Stud. 2016;4:153-9.

[21] . Elliott R, Fox CM, Beltyukova SA, Stone GE, Gunderson J, Zhang X. Deconstructing therapy outcome measurement with rasch analysis of a measure of general clinical distress: the Symptom Checklist-90-Revised. Psychol Assess. 2006;18:359-72. 10.1037/1040-3590.18.4.359 17154757

[22] . Loutsiou-Ladd A, Panayiotou G, Kokkinos CM. A review of the factorial structure of the Brief Symptom Inventory (BSI): Greek evidence. Int J Test. 2008;8:90-110.

[23] . Thomas ML. Rewards of bridging the divide between measurement and clinical theory: demonstration of a bifactor model for the Brief Symptom Inventory. Psychol Assess. 2012;24:101-13. 10.1037/a0024712 21767026

[24] . Urbán R, Arrindell WA, Demetrovics Z, Unoka Z, Timman R. Cross-cultural confirmation of bi-factor models of a symptom distress measure: symptom Checklist-90-Revised in clinical samples. Psychiatry Res. 2016;239:265-74. 10.1016/j.psychres.2016.03.039 27039011

[25] . United Nations Educational, Scientific and Cultural Organization (UNESCO), Institute for Statistics. International Standard Classification of Education: ISCED 2011 [Internet]. 2012 [cited 2021 Sep 6]. uis.unesco.org/sites/default/files/documents/international-standard-classification-of-education-isced-2011-en.pdf

[26] . Rosseel Y. Lavaan: an R package for structural equation modeling and more. Version 0.5--12 (BETA). J Stat Softw. 2012;48:1-36.

[27] . Team RC. R: A language and environment for statistical computing. Version 3.0.1 [software]. 2013. r-project.org/

[28] . Finney SJ, DiStefano C, Kopp JP. Overview of estimation methods and preconditions for their application with structural equation modeling. In: Schweizer K, DiStefano C, editors. Principles and methods of test construction: standards and recent advances. Göttingen: Hogrefe; 2016. p. 135-65.

[29] . DiStefano C. Examining fit with structural equation models. In: Schweizer K, DiStefano C, editors Principles and methods of test construction: standards and recent advances. Göttingen: Hogrefe; 2016. p. 166-93.

[30] . Rodriguez A, Reise SP, Haviland MG. Evaluating bifactor models: calculating and interpreting statistical indices. Psychol Methods. 2016;21:137-50. 10.1037/met0000045 26523435

[31] . Reise SP, Bonifay WE, Haviland MG. Scoring and modeling psychological measures in the presence of multidimensionality. J Pers Assess. 2013;95:129-40. 10.1080/00223891.2012.725437 23030794

[32] . Epskamp S. semPlot: path diagrams and visual analysis of various SEM Packages’ output. R package version 1.1.2 [software]. 2019. CRAN.R-project.org/package=semPlot

[33] . Dueber D. BifactorIndicesCalculator: bifactor indices calculator. R package version 0.2.0 [software]. 2020. CRAN.R-project.org/package=BifactorIndicesCalculator

[34] . The jamovi project. jamovi. Version 1.6 [software]. 2020. www.jamovi.org.

[35] . Pettersson E, Larsson H, D’Onofrio BM, Bölte S, Lichtenstein P. The general factor of psychopathology: a comparison with the general factor of intelligence with respect to magnitude and predictive validity. World Psychiatry. 2020;19:206-13. 10.1002/wps.20763 PMC7215062 32394574

[36] . Wang Y, Duan Z, Ma Z, Mao Y, Li X, Wilson A, et al. Epidemiology of mental health problems among patients with cancer during COVID-19 pandemic. Transl Psychiatr. 2020;10:263. 10.1038/s41398-020-00950-y PMC7393344 32737292

[37] . Ellis WE, Dumas TM, Forbes LM. Physically isolated but socially connected: Psychological adjustment and stress among adolescents during the initial COVID-19 crisis. Can J Behav Sci. 2020;52:177-87.

[38] . Viana MC, Andrade LH. Lifetime prevalence, age and gender distribution and age-of-onset of psychiatric disorders in the São Paulo Metropolitan Area, Brazil: results from the São Paulo Megacity Mental Health Survey. Braz J Psychiatry. 2012;34:249-60. 10.1016/j.rbp.2012.03.001 23429770

[39] . da Silva AG, Miranda DM, Diaz AP, Teles AL, Malloy-Diniz LF, Palha AP. Mental health: why it still matters in the midst of a pandemic. Braz J Psychiatry. 2020;42:229-31. 10.1590/1516-4446-2020-0009 PMC7236155 32267344

